# Effectiveness of Acupuncture for Primary Ovarian Insufficiency: A Systematic Review and Meta-Analysis

**DOI:** 10.1155/2015/842180

**Published:** 2015-05-18

**Authors:** Junyoung Jo, Yoon Jae Lee, Hyangsook Lee

**Affiliations:** ^1^Department of Korean Gynecology, Graduate School, Kyung Hee University, Seoul 130-702, Republic of Korea; ^2^Conmaul Hospital, Seoul 137-881, Republic of Korea; ^3^Department of Korean Gynecology, CHA Bundang Medical Center, CHA University, Seongnam 463-712, Republic of Korea; ^4^Acupuncture and Meridian Science Research Center, College of Korean Medicine, Kyung Hee University, Seoul 130-701, Republic of Korea

## Abstract

*Objective*. This systematic review aimed to assess current evidence from randomized controlled trials (RCTs) on the effects of acupuncture for patients with primary ovarian insufficiency (POI). *Methods*. We searched twelve databases to identify relevant studies published before July 2014. The outcomes were serum follicle-stimulating hormone (FSH) levels and resumption of menstruation. Two reviewers independently assessed the risk of bias using the Cochrane's tool, extracted the results, and evaluated the overall level of the evidence using Grading of Recommendations Assessment, Development, and Evaluation (GRADE) criteria. *Results*. Eight RCTs were selected. Acupuncture significantly lowered serum FSH levels and more women receiving acupuncture reported resumption of menses. However, the results should be interpreted with caution due to a small number of participants, high risk of bias for blinding, and likely publication bias. The level of evidence for FSH level and resumption of menses were assessed as “low” using GRADE. *Conclusion*. The current evidence on acupuncture for POI is insufficient to draw a firm conclusion due to scarcity of studies with a low risk of bias and likely publication bias. Further rigorously designed and conducted studies are needed to confirm the effectiveness and safety of acupuncture in patients with POI.

## 1. Introduction

Primary ovarian insufficiency (POI) is considered to be present when a woman who is less than 40 years old has had oligo/amenorrhea for 4 months or more, with two serum follicle-stimulating hormone (FSH) levels, obtained at least one month apart, in the menopausal range [1]. The incidence of POI is 1-2% of women younger than 40 years of age and 0.1% of women younger than 30 years of age [2]. Although autoimmune disorders, specific gene mutations, and environmental factors may play a role in POI, in most cases, the causes of POI are largely unknown [3]. POI is associated with infertility, which in most cases is due to the failure of follicles in the ovary to respond to stimulation [3].

Women with POI experience menopausal symptoms, such as hot flushes, night sweats, and vaginal dryness, which are similar to those of a natural menopause [4]. However, POI differs from menopause in that there is varying and unpredictable ovarian function in approximately 50% of cases, and about 5 to 10% of women conceive and deliver a child after they have received the diagnosis [3, 5].

Although data from randomized controlled trials (RCTs) are lacking, most experts agree that physiologic estrogen and progestin replacement is a reasonable option in the case of young women with POI [3]. While there are concerns about increased risks of breast cancer, heart attacks, and strokes in menopausal women undergoing hormone replacement therapy (HRT), it is yet to be further investigated whether similar concerns apply to women with POI taking HRT [4].

Acupuncture has been used in eastern Asian countries for thousands of years and suggested as an effective approach to managing vasomotor symptoms [6]. It has been found effective in reducing the hot flush severity in women with breast cancer [7] and in perimenopausal and postmenopausal women [8]. However, there has been no systematic investigation on its therapeutic effects on POI. Therefore, this systematic review aimed at summarizing and evaluating the current evidence from RCTs on the effects of acupuncture with regard to serum hormone levels and recurrence of menstruation for patients with POI.

## 2. Materials and Methods

### 2.1. Search Strategy

We searched electronic databases for relevant studies published before July 16, 2014, comprising four international, three Chinese, two Korean, and three Japanese databases: Ovid-Medline (1946 to July, Week 2, 2014), Ovid-EMBASE (1974 to July 16, 2014), Cochrane Central Register of Controlled Trials (CENTRAL), the Allied and Complementary Medicine Database (AMED, 1985 to July 2014), China National Knowledge Infrastructure (CNKI), Wanfang DATA, Chongqing VIP, KoreaMed, Oriental Medicine Advanced Searching Integrated System (OASIS), Japan Science and Technology Information Aggregator, Electronic (J-STAGE), Medical Online, and Igaku Chuo Zasshi (ICHUSHI). Various combinations of Mesh headings and keywords were used, including “premature ovarian failure,” “primary ovarian insufficiency,” “amenorrhea,” “acupuncture,” and “electroacupuncture.” The detailed searching strategies are provided in appendix. Grey literature searching and hand search were done in bibliographic references in relevant publications (e.g., gynecology textbooks, integrative and complementary and alternative medicine textbooks, grey literature, clinical guidelines of infertility, or other review articles). No language restrictions were imposed.

### 2.2. Study Selection 

#### 2.2.1. Types of Studies

All RCTs evaluating the effects of acupuncture in the treatment of POI were included. Nonrandomized trials, quasi-experimental studies, and observational studies were excluded. Animal studies, qualitative studies, letters, news articles, editorials, and commentaries were also excluded.

#### 2.2.2. Types of Participants

Women who were diagnosed with POI were considered: the diagnostic criteria for POI included women who were less than 40 years old with elevated serum FSH levels (usually above 40 IU/L) detected on at least two separate occasions 1 month apart and oligo/amenorrhea for 4 months or more. Other diseases such as insensitive ovarian syndrome and gonadal dysgenesis were excluded.

#### 2.2.3. Types of Interventions

We included trials in which acupuncture involved the insertion of needles into traditional meridian points regardless of types of acupuncture. Therefore, the studies which did not involve skin penetration, such as acupressure or moxibustion, were excluded. Studies including application of minimal moxibustion on a small number of points were included if acupuncture was defined as a main intervention. Studies investigating the combined effects of acupuncture with other related modalities such as herbal medicine or laser acupuncture were also excluded.

#### 2.2.4. Types of Control Groups

Trials adopting sham acupuncture, no treatment, or other active treatments (e.g., standard treatment like HRT) for a control group were considered. Trials where other treatments were applied to both groups (acupuncture treatment group and control group) in the same manner were also included.

#### 2.2.5. Types of Outcome Measures

Primary outcomes were serum FSH levels and resumption of menstruation. Secondary outcomes were serum hormone levels of luteinizing hormone (LH) and estradiol (E2) and menopausal symptoms.

All studies were reviewed and selected independently by two reviewers (Junyoung Jo and Yoon Jae Lee). The titles and abstracts were reviewed and articles which did not fit the eligibility criteria were excluded. If the title or abstract appeared to meet the eligibility criteria or we could not determine its eligibility, the full texts of the articles were obtained for further evaluation. Discrepancies between the reviewers were resolved by consensus among all three reviewers.

### 2.3. Data Extraction

Two independent reviewers (Junyoung Jo and Yoon Jae Lee) extracted data using a standardized data extraction form. Any discrepancies were resolved by consensus or consultation with another reviewer (Hyangsook Lee). The characteristics and general information (inclusion/exclusion criteria of participants, acupuncture intervention, comparison group, outcomes, adverse events (AEs), and follow-up period) were extracted and tabulated.

### 2.4. Risk of Bias Assessment

Two authors (Junyoung Jo and Yoon Jae Lee) independently evaluated the risk of bias of the included studies. The risk of bias was assessed using the risk of bias assessment tool by the Cochrane Collaboration [9]. The criteria consist of 7 items related to selection bias (random sequence generation and allocation concealment), performance bias (blinding of participants and personnel), detection bias (blinding of outcome assessment), attrition bias (incomplete outcome data), reporting bias (selective outcome reporting), and other sources of bias. Any discrepancies between the two reviewers were resolved by a discussion with a corresponding author (HL) until consensus was reached.

### 2.5. Data Synthesis

Statistical analyses were performed with the Review Manager program (Version 5.3 Copenhagen: The Nordic Cochrane Centre, The Cochrane Collaboration, 2014). Trials were combined according to the type of intervention, type of outcome measure, and/or control. The results were pooled using a Mantel-Haenszel random effects model and expressed as risk ratios (RR) for the dichotomous variable (resumption of menstruation). For the continuous variables (hormonal outcomes), the results were pooled and expressed as mean differences (MD) using inverse variance methods and the random-effects model with 95% confidence intervals (CI). Heterogeneity among studies was assessed using Cochrane's *Q* and *I* ^2^ statistic [10]. The *I* ^2^ statistic indicates the proportion of variability among trials that is not explained by chance alone and we considered an *I* ^2^ value of more than 50% to indicate a substantial heterogeneity [10, 11]. If a substantial heterogeneity was detected, we explored the reasons for heterogeneity. When there were more than 10 trials in the analysis, reporting biases such as publication bias were assessed by funnel plots. If asymmetry is suggested by a visual inspection, we performed exploratory analyses to investigate it using Egger's method [10]. Subgroup analyses were tried according to types of intervention and follow-up period, taking into consideration the characteristics of the included studies.

### 2.6. Level of Evidence

The Grades of Recommendations, Assessment, Development, and Evaluation (GRADE) were used to assess the level of evidence and summarize each outcome. The GRADE is a method of grading the level of evidence developed by the GRADE Working Group [12, 13]. The GRADEpro software (version 3.6 for Windows, Grade Working Group) was used.

## 3. Results

### 3.1. Results of the Search

**Figure 1:**
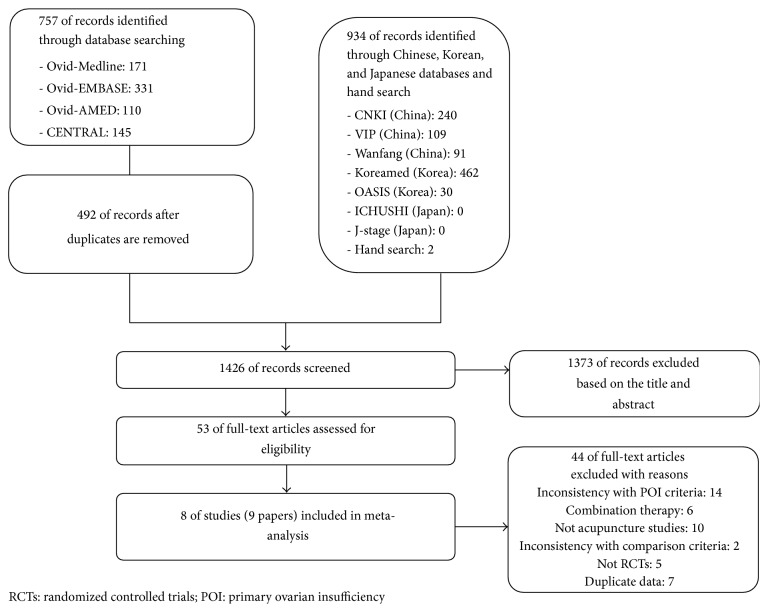
Flow diagram of searching and article selection.

Our initial search identified and screened 1426 articles. We excluded 1373 articles based on the title and abstract and retrieved 53 articles for more detailed evaluation. From these, we excluded 44 publications and included 8 studies (9 publications) in our review (Figure 1).

### 3.2. Included Studies

Eight RCTs with 620 participants met the inclusion criteria. All of them were parallel-group RCTs, originated from China and published in Chinese. One article [14] was a master's thesis not published in peer-reviewed journal and 7 were published in peer-reviewed journals.

### 3.3. Participants

The diagnostic criteria for POI were not clearly reported in some studies [15–18]. The participants' mean age and duration of amenorrhea were not clearly reported in 5 [14, 15, 19–21] and 4 trials [14, 15, 20, 21], respectively. Jia and Duan [15] did not report the hormone levels in POI diagnostic criteria. Yang et al. [16], Sha et al. [17], and Wang et al. [18] did not report exactly if they had tested serum FSH levels twice.

### 3.4. Interventions

Four trials [14, 16, 17, 21] tested acupuncture therapy alone in the treatment group. Fu [14] used acupuncture with placebo Chinese herbal medicine (CHM), so we considered the intervention as acupuncture therapy alone. The others used acupuncture therapy combined with CHM [18] or HRT [15, 20, 22] in the treatment group. Four trials [16–18, 22] used manual acupuncture, two [14, 15] used electroacupuncture, and the other two [20, 21] used acupoint catgut implantation. Acupuncture interventions varied in acupoint selection, frequency of treatment, and number of treatments among the studies. The duration of therapy ranged from 3 to 6 months. The characteristics of the included studies are presented in Table 1.

### 3.5. Risk of Bias in Included Studies

**Figure 2:**
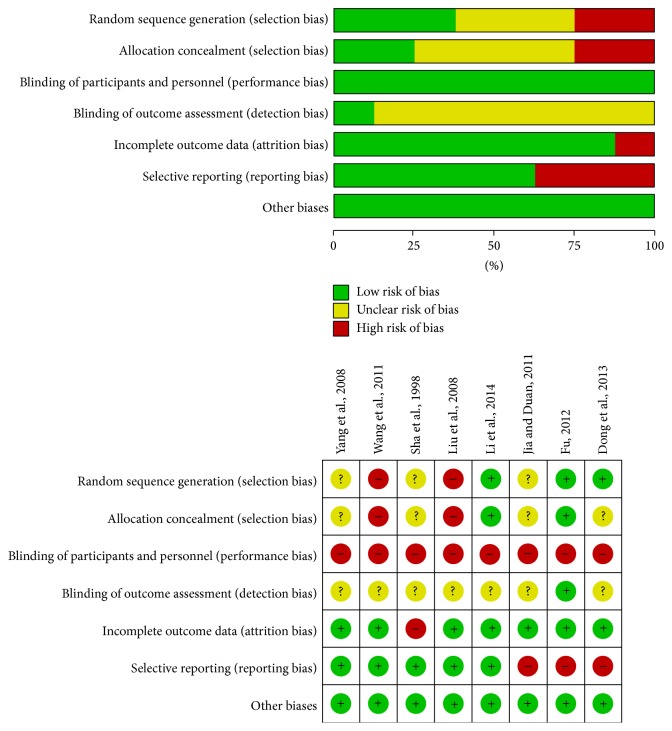
Risk of bias of the included studies.

Among the 8 included RCTs, two studies [18, 21] adopted inadequate randomization procedures, while three [14, 20, 22] described adequate methods of random sequence generation: one study [18] adopted sequence generated by rule based on order of treatment and the other [21] used sequence generated by rule based on odd or even date of admission. We rated the other three studies [15–17] as having an unclear risk of bias because they failed to describe an adequate method of random number generation. Two trials [14, 20] were at low risk of bias for allocation concealment, while two [18, 21] were at high risk of bias. We rated the other 4 studies [15–17, 22] as having an unclear risk of bias because they did not describe an acceptable method of allocation concealment. For participant and outcome assessment blinding, we gave a high risk of bias to all trials because participants were not blinded. Only one trial [14] blinded outcome assessors. The other seven trials [15–18, 20–22] did not report any information on blinding of outcome assessors. One trial [17] was at high risk of bias for incomplete outcome data as missing data in some hormones accounted for more than 20% of the participants with reasons not provided. The other seven trials [14–16, 18, 20–22] were low risk of bias. Three trials [14, 15, 22] were at high risk of bias for selecting reporting. Jia and Duan [15] did not report FSH, E2, and LH outcomes. Dong et al. [19, 22] reported the incomplete data of Kupperman index. Fu [14] presented incomplete data of hormone outcomes. The other five trials [16–18, 20, 21] were at low risk of bias. Other sources of bias were at low risk in all of the included studies. A graphical summary of the risks of bias assessment is presented in Figure 2.

### 3.6. Effects of Acupuncture

#### 3.6.1. Primary Outcomes

**Figure 3:**
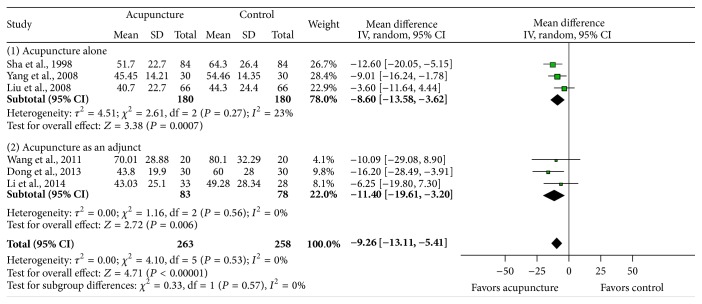
The impact of acupuncture on follicle-stimulating hormone (FSH) level.

*Effects on FSH*. Six studies [16–18, 20–22] evaluated FSH levels at the end of treatment. The pooled results showed a significant decrease in the FSH level in the acupuncture group compared with the control groups (MD −9.26, 95% CI: −13.11–−5.41, *I* ^2^ = 0%, *P* < 0.00001). The direction of effectiveness was the same in all studies (Figure 3).

**Figure 4:**
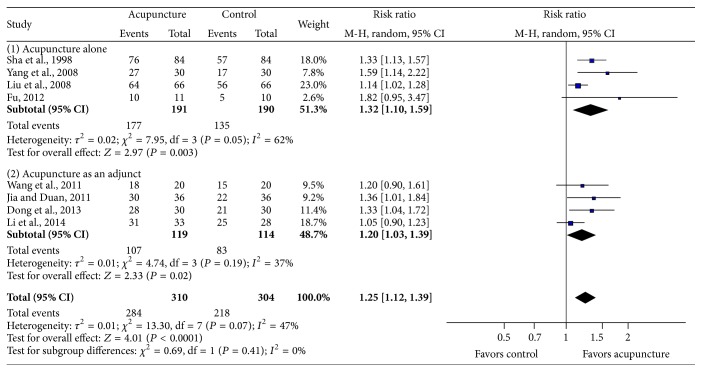
The impact of acupuncture on resumption of menstruation.

*Effects on Resumption of Menstruation*. Resumption of menstruation was reported in its own criteria in each study. We defined resumption of menstruation if the menstruation recovered at least once after treatment. The pooled data showed that significantly more women resumed menstruation after acupuncture treatment than those in the control group (Figure 4, RR 1.25, 95% CI: 1.12–1.39, *I* ^2^ = 47%, *P* < 0.0001).

#### 3.6.2. Secondary Outcomes

**Figure 5:**
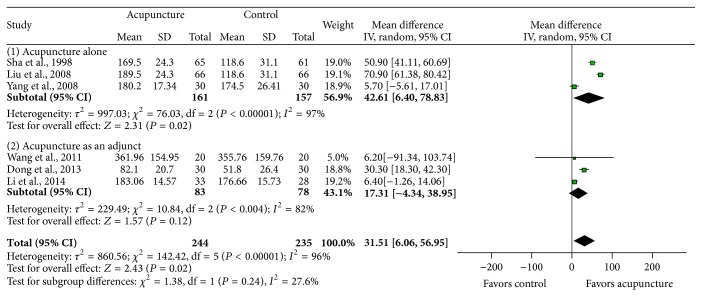
The impact of acupuncture on estradiol (E2) level.

*Effects on E2*. The results of E2 were extracted in 6 studies [16–18, 20–22]. The pooled results showed a significant difference between acupuncture and control groups (MD 31.51, 95% CI: 6.06–56.95, *P* = 0.02) in Figure 5. But there was considerable heterogeneity (*I* ^2^ = 96%). The studies of acupuncture alone [16, 17, 21] found a significant difference between the groups, while the studies of acupuncture with other treatments [18, 20, 22] found no significant difference. We conducted subgroup analysis according to the control group, intervention type (e.g., electroacupuncture and catgut implantation), or follow-up periods, but heterogeneity was not resolved (data not shown).

**Figure 6:**
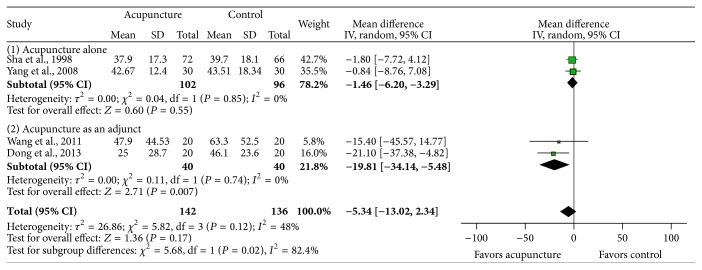
The impact of acupuncture on luteinizing hormone (LH) level.

*Effects on LH*. The pooled results from 4 trials [16–18, 22] showed no significant difference in LH levels between the treatment and control groups (MD −5.34, 95% CI: −13.02–2.34, *I* ^2^ = 48%, *P* = 0.17) using inverse variance method in random-effect model. The acupuncture treatment showed a tendency for decrease in the levels of LH but did not reach a statistical significance (Figure 6). Acupuncture significantly lowered LH level when added to HRT or herbal medicine [18, 22], while acupuncture alone found no significant difference [16, 17].

*Effects on Menopausal Symptoms (Kupperman Index)*. Although the outcomes related with perimenopausal symptoms were reported in all included studies, they evaluated the symptoms with different tools; thus we did not pool the data. Only two studies [19, 20] used Kupperman index. Dong et al. [19] reported that mean Kupperman index score was reduced in both groups, from 16.65 to 5.43 in the acupuncture combined with HRT group and from 16.83 to 11.90 in the HRT only group. Li et al. [20] reported that the Kupperman index was reduced significantly in both groups after treatment. But after 6 months from the final treatment, there was a significant difference between acupuncture group and control group. The effect was maintained in the acupuncture group but was not so in the control group at 6 months follow-up [20].

*Adverse Events*. Of the 8 studies, only two studies [20, 21] reported AEs and the rest of them did not mention AEs at all. Liu et al. [21] mentioned that there were no serious AEs reported. Li et al. [20] reported that there were only mild AEs including bruises, induration, and mild edema which did not need treatment.

#### 3.6.3. Level of Evidence

The levels of evidence as determined by GRADE were from very low to low (Table 2). Most of the studies did not report blinding, randomization sequence generation, or allocation concealment methods, so all outcomes were initially downgraded. The inconsistency domain was downgraded for the outcome of E2. In addition, the imprecision domain of all outcomes was downgraded due to small participants of all outcomes.

## 4. Discussion

### 4.1. Summary of Main Findings

This systematic review and meta-analysis has shown that acupuncture may reduce serum FSH levels, increase E2 levels, and restore the menstruation in patients with POI, while LH levels were not significantly altered by acupuncture. However, these seemingly positive results should be interpreted with caution mainly due to a small number of participants, high risk of bias for blinding, and the probability of studies with negative results left unpublished. The level of evidence of FSH, resumption of menstruation, and LH was assessed as “low” using GRADE. The level of evidence of E2 and symptoms was “very low” due to serious risk of bias and inconsistency. Acupuncture treatment seems to be associated with few AEs in women with POI, but the evidence is limited due to poor reporting and only two out of 8 studies reported such data.

### 4.2. Potential Mechanism of Acupuncture in Treating POI

Acupuncture has been suggested as an effective management option for vasomotor symptoms [6]. It is also increasingly being used in reproductive medicine including improving pregnancy outcomes of in vitro fertilization (IVF) treatment and management of ovulation disorders [23]. Zheng et al. [24] reported that acupuncture could be effective in improving pregnancy outcomes in women undergoing IVF in their systematic review. Acupuncture treatments also resulted in higher ovulation frequency in lean/overweight women with polycystic ovary syndrome [25]. Although the therapeutic mechanisms of acupuncture in the reproductive disorders are yet to be fully established, it is suggested that acupuncture can modulate hypothalamic-pituitary-ovary axis (HPOA) [26]. Besides the HPOA hypothesis, modulation of autonomic nervous function and increasing ovarian blood flow are also suggested as the mechanism of acupuncture effects on gynecological disorders [26, 27]. Further studies will elucidate the underlying mechanism of acupuncture treatment to establish its role in obstetrics and gynecology [28, 29].

### 4.3. Applicability of the Current Evidence

This systematic review included a total of eight RCTs that evaluated acupuncture in the treatment of POI. We found that acupuncture significantly restored the menstruation as well as improvement in hormone levels.

The pooled data on serum FSH and E2 suggest that acupuncture could be effective to patients with POI. It showed that acupuncture treatment increased serum E2 (MD 31.51, 95% CI: 6.06–56.95, *P* = 0.02) levels and reduced serum FSH (MD −9.26, 95% CI: −13.11–−5.41, *P* < 0.00001) levels. These results are similar with Wu et al.'s [30] study: they reported that CHM might relieve symptoms of POI partly through increasing E2 levels (MD 22.00, 95% CI: 2.90–41.10, *P* = 0.024) and thereby decreasing serum FSH levels (MD −6.59, 95% CI: −9.06 to–−4.12, *P* < 0.001) in patients with POI. There were no significant differences in LH levels in both studies.

We also found that acupuncture could help resumption of menstruation in women with POI. Significantly more women receiving acupuncture treatment resumed menstruation compared with those in the control groups (RR 1.25, 95% CI: 1.12–1.39, *I* ^2^ = 47%, *P* < 0.0001). This finding needs to be confirmed in future trials with ovulation detection. Acupuncture treatments also relieved perimenopausal symptoms in all included studies.

While this finding seems promising, it should be interpreted with caution because of the small number of included studies and participants. In the 8 included studies, participants ranged only from 23 to 168 in each trial (13 to 84 patients in the acupuncture group versus 10 to 84 patients in the control group). In addition, no trial reported a formal sample size calculation, which is essential for ensuring adequate statistical power.

As is usual with other systematic reviews on acupuncture, one limitation of this systematic review lies in the clinical and methodological diversity of the included studies. There was a considerable heterogeneity across trials in terms of participants, the tested acupuncture interventions, control procedures, and the outcome measurements. These clinical and methodological diversities may have yielded considerable heterogeneity in our meta-analyses, making their generalizability more complicated.

In addition, there are issues about high risk of bias and unavoidable placebo effect in the included trials. The overall estimate of the intervention effect can be exaggerated to a substantial degree when there is inadequate allocation concealment [31] or lack of blinding in trials where a subjective outcome is analyzed [32]. Also, no studies adopted a sham acupuncture control group, so it is not possible to rule out placebo response in our review.

However, a recent review on placebo effects suggested that biochemical parameters such as growth hormone and cortisol are less placebo-sensitive than physical ones, for example, gastric and pulmonary function [33]. In our review, we assessed the serum hormone levels which belong to biochemical outcomes, so we need to consider the results in this respect.

### 4.4. The Safety of Acupuncture in Treating POI

In this review, only two [20, 21] out of 8 studies reported information about AEs. The other studies did not mention AEs at all. The absence of information on AEs does not mean that the intervention is safe [34]. So, we cannot assure the safety of acupuncture in patients with POI. Although existing literatures on acupuncture in a variety conditions have supported that it is a relatively safe treatment modality, acupuncture treatment itself varies considerably and is not without risks [35]. Future clinical trials are required to report AEs with more explanations [36].

### 4.5. Implications for Further Studies and Clinical Applications

To confirm the ovarian activity, assessments should be conducted more rigorously in future trials. In this review, all studies did not document detailed information on hormone measurement. Hormone levels could vary according to the measurement time during menstrual cycle. Also, resumption of menstrual cycles should be clearly defined (e.g., at least two consecutive episodes of uterine bleeding within 3 to 6 weeks) [5]. Serum E2 levels of >184 pmol/L could be used as the indicator of follicular activity because women with absent or nonfunctioning follicles typically produce less E2 [37].

Although acupuncture seems to be beneficial to control sex hormones and resumption of menstruation, it was not enough to make the hormone levels into normal ranges. In our review, duration of amenorrhea reported in a half of included studies varied from 2.3 to 5.6 years in patients with POI. Bidet et al. [5] reported that seventy-six (88%) patients experienced intermittent ovarian function during the first year after POI diagnosis and only three patients after 4 years. In consideration of duration of amenorrhea, the effects of acupuncture seem to be promising in this review.

All studies were conducted relatively short period and were not followed up for long term. Therefore, the effect related pregnancy rate could not be confirmed. More studies with long term follow-up are needed for verifying of pregnancy rate and sustainability of menstruation.

POI encompasses a heterogeneous spectrum of conditions, with phenotypic variability among patients [38]. Although POI is most frequently idiopathic or caused by autoimmune disorders, genetic causes, and chromosomal abnormalities or caused by radiotherapy and/or chemotherapy of cancer, most of included studies did not report the screening data or exclusion criteria. Future trials should clearly define inclusion and exclusion criteria.

Most of the included studies either improperly reported or did not report items such as type of randomization, allocation concealment, blinding, and detailed acupuncture procedures. Future trials should improve their reporting quality. Following the Consolidated Standards of Reporting Trials (CONSORT) statement [39] and the Standards for Reporting Interventions in Clinical Trials of Acupuncture (STRICTA) [40] will improve quality of a study by reporting detailed information.

This review is the first systematic review and meta-analysis of current relevant RCTs of acupuncture treatments for POI patients and it may provide basis for further studies. Robles et al. [41] showed that there was no scientific evidence that any treatment can improve the ovulation and pregnancy rates in patients with POI. Ben-Nagi and Panay [42] suggested that it is important to normalize gonadotropin levels for successful conception. In this context, acupuncture may have a potential for women with POI.

## 5. Conclusion

This systematic review and meta-analysis suggests that current evidence showing that acupuncture for restoration of menstruation as well as improvement in hormone levels in patients with POI is insufficient to make a firm conclusion due to a lack of studies with a low risk of bias. Further rigorously designed studies are needed to confirm the effectiveness and safety of acupuncture in patients with POI.

## Supplementary Material

Supplementary Material: Search strategies are provided in Appendix 1.

## Figures and Tables

**Table 1:** The characteristics of the included studies.

Author/year	Sample size (Mean age, range)	Diagnostic criteria	Intervention	Treatment session/period	Acupoints	Comparison	Outcome measures	Outcome assessment
Dong et al., 2013 [19, 22]	60 Acupuncture: NR, 24–36 Control: NR, 23–36	Age <40, amenorrhea ≥6 months, FSH ≥40 U/L, and E2 <73 pmol/L	Acupuncture and HRT	Up to 6 sessions over 6 months (15 treatments/session)	CV6, CV4, SP6, BL7, SP10, LI4, LR3, KI3, BL23, BL15, BL20	HRT	FSH, E2, Kupperman index, recovery of menstruation	After treatment (6 months)

Wang et al., 2011 [18]	40 Acupuncture: 35.4, 26–44 Control: 34.9, 24–43	Age <40, amenorrhea ≥6 months, FSH >40 mIU/mL, LH >40 mIU/mL, and E2 <30 pg/mL	Acupuncture and CHM	Up to 6 sessions over 3 months (10 treatments/session)	BL20, BL21, BL18, BL23, CV4, CV2, SP10, Ex-CA1, SP6	CHM	E2, FSH, LH, menopausal symptoms	After treatment (3 months)

Fu, 2012 [14]	23 Acupuncture: NR, 25–40 Control: NR, 25–40	Age <40, amenorrhea ≥6 months, FSH >40 IU/L, LH >30 IU/L, and E2 <25 ng/mL	EA and placebo CHM	20 treatments over 3 months (4/week for first 2 weeks; 2/week for 3-4 weeks; weekly for 5–12 weeks)	Bilateral ST25 and BL33	HRT and placebo CHM	E2, FSH, LH, recovery of menstruation	After treatment (3 months)

Sha et al., 1998 [17]	168 (35.5, 26–40)	Prolactin normal, FSH >40 *µ*g/L	Acupuncture^a^	Up to 6 sessions (1 session: 20 treatments)	CV4, CV3, KI12, Ex-CA1, BL23 SP6, SP9, BL18, HT6, KI7 added in some cases BL19, GV4, BL32, SP8 added in some cases	Clomiphene and estrogen	E2, FSH, LH	After treatment (6, 7, and 9 months)

Yang et al., 2008 [16]	60 (35.5, 28–40)	Age <40, amenorrhea ≥4 months, FSH >40 U/L, LH ≥normal, and E2 <73.2 pmol/L	Acupuncture A group: liver-kidney yin deficiency pattern identification of TCM B group: spleen-kidney yang deficiency pattern	90 treatments	A group: CV4, ST30, Ex-CA1, CV3, SP6, ST36, SP10, LR3, KI3 B group: BL17, BL18, BL19, BL23, BL26, BL32	HRT	E2, FSH, LH, recovery of menstruation	After treatment (3 months)

Li et al., 2014 [20]	65 (NR, 18–40)	Age <40, amenorrhea ≥6 months, FSH >40 IU/L, E2 <100 pmol/L, and menopausal symptoms	Acupoint catgut implantation and HRT	Once/2-3 weeks for 6 months	Major points: (1) PC6, ST36 (2) CV4, SP6 Selected points: BL23, BL19, CV6, BL21, Ex-CA1, GV4, CV7, CV3, BL26, BL32, ST40	HRT	Kupperman index, FSH, E2	After treatment (6 months) and follow-up at 12 months about symptoms

Liu et al., 2008 [21]	132 (NR)	Age <40, amenorrhea ≥4 months, FSH >40 IU/L, and E2 <73.2 pmol/L	Acupoint catgut implantation	Total 8 treatments over 6 months	BL18, BL20, BL23, LR14, LR13, GB25	HRT	FSH, E2, recovery of menstruation	After treatment (6 months) and follow-up at 12 months about symptoms

Jia and Duan, 2011 [15]	23 Acupuncture: NR, 33–40 Control: NR, 32–38	Age <40, amenorrhea, hypergonadotropic hypogonadism ≥6 months	EA and HRT	Once daily but stopped after recovery of menstruation	Auricular points (Shenmen, internal genitalia, endocrine, pituitary) CV6, CV4, ST30, BL18, BL23, LI4, SP10, ST30, ST36, SP6	HRT	E2, FSH, LH, recovery of menstruation	After treatment (6 months)

CHM: Chinese herbal medicine; EA: electroacupuncture; E2: serum estradiol; FSH: follicle-stimulating hormone; HRT: hormone replacement therapy; LH: luteinizing hormone; TCM: traditional Chinese medicine; NR: not reported.

^a^Warm needling and cupping added in some cases.

**Table 2:** Level of evidence (GRADE).

Outcomes	Number of participants (number of studies)	Illustrative comparative risks (95% CI)	Level of evidence
FSH	521 (6 studies)	The mean FSH in acupuncture groups was 9.26 lower (13.11 to 5.41 lower)	Low *⊕⊕⊖⊖*

Resumption of menstruation	614 (8 studies)	Acupuncture group: 918 per 1000 (873 to 948) Control group: 907 per 1000 (857 to 940)	Low *⊕⊕⊖⊖*

Estradiol (E2)	479 (6 studies)	The mean E2 in the acupuncture group was 31.51 higher (6.06 to 56.95 higher)	Very low *⊕⊖⊖⊖*

LH	278 (4 studies)	The mean LH in the acupuncture groups was 5.34 lower (13.02 lower to 2.34 higher)	Low *⊕⊕⊖⊖*

Symptoms (Kupperman index)	121 (2 studies)	The mean symptom score in the acupuncture group ranged from 11.22 to 12.1 higher.	Very low *⊕⊖⊖⊖*
