# Supplementary material for: Effectiveness of Acupuncture for Primary Ovarian Insufficiency: A Systematic Review and Meta-Analysis
Source: Evid Based Complement Alternat Med. 2015 May 18;2015:842180. doi: 10.1155/2015/842180 (PMC4451156; doi:10.1155/2015/842180)
Supplement: Supplementary file 1 — Supplementary Material: Search strategies are provided in Appendix 1. [file 842180.f1.docx]

Appendix 1 Search Strategy

|  | Ovid MEDLINE(R) 1946 to July Week 2 2014 | Date : July 16. 2014 | | |
| --- | --- | --- | --- | --- |
|  | Searches | Results | | |
| 1 | exp Primary Ovarian Insufficiency/ | 1724 | | |
| 2 | (ovar* and (failure or function or damage)).mp. | 31016 | | |
| 3 | primary ovar* insufficiency.mp. | 1801 | | |
| 4 | premature ovar* failure.mp. | 1681 | | |
| 5 | premature ovar* dysfunction.mp. | 1 | | |
| 6 | premature menopause.mp. | 498 | | |
| 7 | exp Amenorrhea/ | 9038 | | |
| 8 | amenorrhea.tw. | 8517 | | |
| 9 | exp Menstruation Disturbances/ | 24286 | | |
| 10 | OR/1-9 | 57963 | | |
| 11 | exp Acupuncture/ | 1218 | | |
| 12 | acupuncture.tw. | 13314 | | |
| 13 | exp Acupuncture, Ear/ | 261 | | |
| 14 | exp Acupuncture Points/ | 3921 | | |
| 15 | exp Acupuncture Therapy/ | 16640 | | |
| 16 | exp Acupressure/ | 463 | | |
| 17 | acupressure.tw. | 13314 | | |
| 18 | exp Electroacupuncture/ | 2509 | | |
| 19 | electroacupuncture.tw. | 2346 | | |
| 20 | exp Medicine, Chinese Traditional/ | 12134 | | |
| 21 | exp Medicine, Korean Traditional/ | 214 | | |
| 22 | exp Medicine, East Asian Traditional/ | 14345 | | |
| 23 | or/11-22 | 31699 | | |
| 24 | randomized controlled trial.pt. | 378583 | | |
| 25 | controlled clinical trial.pt. | 88802 | | |
| 26 | randomized.ab. | 276556 | | |
| 27 | placebo.ab. | 147643 | | |
| 28 | drug therapy.fs. | 1717215 | | |
| 29 | randomly.ab. | 195852 | | |
| 30 | trial.ab. | 286811 | | |
| 31 | groups.ab. | 1257501 | | |
| 32 | or/24-31 | | | 3225927 |
| 33 | exp animals/ not humans.sh. | | | 3968663 |
| 34 | 32 NOT 33 | | | 2745937 |
| 35 | 10 AND 23 AND 34 | | 171 | |

|  | Ovid EMBASE 1974 to July 16 2014 | Date : July 16. 2014 | |
| --- | --- | --- | --- |
|  | Searches | Results | |
| 1 | exp premature ovarian failure/ | | 2340 |
| 2 | (ovar* and (failure or function or damage)).mp. | | 52572 |
| 3 | primary ovar* insufficiency.mp. | | 366 |
| 4 | premature ovar* failure.mp. | | 3436 |
| 5 | premature ovar* dysfunction.mp. | | 7 |
| 6 | premature menopause.mp. | | 800 |
| 7 | exp Amenorrhea/ | | 17125 |
| 8 | amenorrhea.tw. | | 10626 |
| 9 | exp menstruation disorder/ | | 51560 |
| 10 | OR/1-9 | | 101469 |
| 11 | exp Acupuncture/ | | 32733 |
| 12 | acupuncture.tw. | | 21449 |
| 13 | exp Acupressure/ | | 1292 |
| 14 | acupressure.tw. | | 781 |
| 15 | exp Electroacupuncture/ | | 4079 |
| 16 | electroacupuncture.tw. | | 3264 |
| 17 | exp Chinese medicine/ | | 23911 |
| 18 | exp Korean medicine/ | | 284 |
| 19 | exp oriental medicine/ | | 2589 |
| 20 | exp acupuncture needle/ | | 234 |
| 21 | OR/11-20 | | 55378 |
| 22 | Clinical trial/ | | 836675 |
| 23 | Randomized controlled trial/ | | 348217 |
| 24 | Randomization/ | | 62585 |
| 25 | Single blind procedure/ | | 18545 |
| 26 | Double blind procedure/ | | 116824 |
| 27 | Crossover procedure/ | | 39521 |
| 28 | Placebo/ | | 255202 |
| 29 | Randomi?ed controlled trial$.tw. | | 100717 |
| 30 | Rct.tw. | | 14297 |
| 31 | Random allocation.tw. | | 1360 |
| 32 | Randomly allocated.tw. | | 20655 |
| 33 | Allocated randomly.tw. | | 1953 |
| 34 | (allocated adj2 random).tw. | | 795 |
| 35 | Single blind$.tw. | | 14654 |
| 36 | Double blind$.tw. | | 148086 |
| 37 | ((treble or triple) adj blind$).tw. | | 399 |
| 38 | Prospective study/ | | 255982 |
| 39 | OR/22-38 | | 1388067 |
| 40 | Case study/ | | 26877 |
| 41 | Case report.tw. | | 269666 |
| 42 | Abstract report/ or letter/ | | 914696 |
| 43 | 40 or 41 or 42 | | 1205547 |
| 44 | 39 NOT 43 | | 1349832 |
| 45 | 10 AND 21 AND 44 | | 331 |

|  | AMED (Allied and Complementary Medicine) 1985 to July 2014 | Date : July 16. 2014 |
| --- | --- | --- |
|  | Searches | Results |
| 1 | (ovar* and (failure or function or damage)).mp. | 44 |
| 2 | premature menopause.mp. | 2 |
| 3 | premature ovar* failure.mp. | 5 |
| 4 | primary ovar* insufficiency.mp. | 0 |
| 5 | premature ovar* dysfunction.mp. | 0 |
| 6 | primary ovar* failure.mp. | 0 |
| 7 | exp Amenorrhea/ | 34 |
| 8 | exp menstruation disorder/ | 420 |
| 9 | amenorrhea.tw. | 73 |
| 10 | OR/1-9 | 463 |
| 11 | exp Acupuncture/ | 3221 |
| 12 | acupuncture.tw. | 8985 |
| 13 | exp Acupressure/ | 272 |
| 14 | acupressure.tw. | 349 |
| 15 | exp Electroacupuncture/ | 748 |
| 16 | electroacupuncture.tw. | 885 |
| 17 | exp Traditional medicine chinese/ | 5412 |
| 18 | exp Acupuncture therapy/ | 6925 |
| 19 | exp Ear acupuncture/ | 390 |
| 20 | or/11-19 | 13036 |
| 21 | 10 AND 20 | 110 |

|  | Cochrane Library | Date : July 16. 2014 |
| --- | --- | --- |
|  | Searches | Results |
| #1 | MeSH descriptor: [Primary Ovarian Insufficiency] explode all trees | 77 |
| #2 | (ovar* and (failure or function or damage)) | 1493 |
| #3 | premature menopause | 129 |
| #4 | premature ovar* failure | 172 |
| #5 | primary ovar* insufficiency | 136 |
| #6 | premature ovar* dysfunction | 57 |
| #7 | primary ovar* failure | 429 |
| #8 | MeSH descriptor: [Amenorrhea] explode all trees | 256 |
| #9 | amenorrhea | 766 |
| #10 | MeSH descriptor: [Menstruation Disturbances] explode all trees | 1465 |
| #11 | #1 or #2 or #3 or #4 or #5 or #6 or #7 or #8 or #9 or #10 | 3379 |
| #12 | MeSH descriptor: [Acupuncture] explode all trees | 150 |
| #13 | MeSH descriptor: [Acupressure] explode all trees | 226 |
| #14 | MeSH descriptor: [Electroacupuncture] explode all trees | 471 |
| #15 | MeSH descriptor: [Acupuncture Therapy] explode all trees | 3078 |
| #16 | MeSH descriptor: [Acupuncture Points] explode all trees | 1051 |
| #17 | MeSH descriptor: [Acupuncture, Ear] explode all trees | 121 |
| #18 | acupuncture | 8613 |
| #19 | acupressure | 569 |
| #20 | electroacupuncture | 1077 |
| #21 | MeSH descriptor: [Medicine, Chinese Traditional] explode all trees | 818 |
| #22 | MeSH descriptor: [Medicine, Korean Traditional] explode all trees | 890 |
| #23 | MeSH descriptor: [Medicine, East Asian Traditional] explode all trees | 16 |
| #24 | #12 or #13 or #14 or #15 or #16 or #17 or #18 or #19 or #20 or #21 or #22 or #23 | 9632 |
| #25 | #11 and #24 in Trials | 145 |

|  | ICHUSHI | Date : July 16. 2014 |
| --- | --- | --- |
|  | Searches | Results |
| #1 | ((鍼療法/TH or acupuncture/AL) or (鍼灸医学/TH or acupuncture/AL)) | 14,917 |
| #2 | (鍼療法/TH or 鍼/AL) | 27,919 |
| #3 | (卵巣不全-早期/TH or 早発卵巣不全/AL) | 332 |
| #4 | (卵巣不全-早期/TH or 卵巣不全-早期/AL) | 220 |
| #5 | (閉経-早発/TH or 早発閉経/AL) | 256 |
| #6 | (卵巣不全-早期/TH or 早発性卵巣機能不全/AL) | 223 |
| #7 | (閉経-早発/TH or 閉経-早発/AL) | 213 |
| #8 | premature/AL and ovarian/AL and failure/AL | 81 |
| #9 | #3 or #4 or #5 or #6 or #7 or #8 | 594 |
| #10 | #1 or #2 | 28,035 |
| #11 | #9 and #10 | 0 |

|  | J-Stage | Date : July 16. 2014 |
| --- | --- | --- |
|  | Searches | Results |
| #1 | 早発卵巣不全 AND 鍼 | 0 |
| #2 | 早発閉経 AND 鍼 | 0 |
| #3 | 早発性卵巣機能不全 AND 鍼 | 0 |

|  | Medical Online | Date : July 16. 2014 |
| --- | --- | --- |
|  | Searches | Results |
| #1 | 早発卵巣不全 AND 鍼 | 0 |
| #2 | 早発閉経 AND 鍼 | 0 |
| #3 | 早発性卵巣機能不全 AND 鍼 | 0 |

|  | CNKI | Date : July 30. 2014 |
| --- | --- | --- |
|  | Searches | Results |
| #1 | (SU='针刺' or SU='电针' or SU='耳针'or SU='针灸'or SU='针'or SU='温针灸'or SU='腹针'or SU='艾灸') and (SU='卵巢早衰'or SU='卵巢功能不全'or SU='卵巢功能早衰'or SU='闭经'or SU='早绝经') | 240 |

|  | VIP | Date : July 30. 2014 |
| --- | --- | --- |
|  | Searches | Results |
| #1 | m=(卵巢功能不全+卵巢早衰+卵巢功能早衰+闭经+早绝经)*m=(针刺+针灸+艾灸+温针灸+电针+腹针+耳针+芒针+针) | 109 |

|  | Wanfang | Date : July 30. 2014 |
| --- | --- | --- |
|  | Searches | Results |
| #1 | (题名或关键词:(卵巢功能不全) + 题名或关键词:(卵巢早衰)+题名或关键词:(卵巢功能早衰)+题名或关键词:(早绝经)+题名或关键词:(闭经)) * 题名或关键词:(针刺+针灸+艾灸+温针灸+电针+腹针+耳针+芒针+针) | 91 |

|  | OASIS | Date : Aug. 29. 2014 |
| --- | --- | --- |
|  | Searches | Results |
| #1 | 난소기능부전 | 1 |
| #2 | 조기폐경 | 0 |
| #3 | 조기난소부전 | 6 |
| #4 | 원발성 난소부전 | 0 |
| #5 | 무월경 | 18 |
| #6 | 월경 이상 | 5 |

|  | Koreamed | Date : Aug. 29. 2014 |
| --- | --- | --- |
|  | Searches | Results |
| #1 | Primary Ovarian Insufficiency | 50 |
| #2 | premature menopause | 19 |
| #3 | primary ovarian failure | 55 |
| #4 | premature ovarian failure | 48 |
| #5 | amenorrhea | 282 |
| #6 | Menstruation Disturbance | 8 |
